# World Congress for NeuroRehabilitation (WCNR) 2022 Vienna: new perspectives in neurorehabilitation

**DOI:** 10.25122/jml-2022-1033

**Published:** 2022-12

**Authors:** Stefana-Andrada Dobran, Andreea Doria Constantinescu, Alexandra Gherman, Dafin Muresanu

**Affiliations:** 1 RoNeuro Institute for Neurological Research and Diagnostic, Cluj-Napoca, Romania; 2 Sociology Department, Babes-Bolyai University, Cluj-Napoca, Romania; 3 Department of Neuroscience, Iuliu Hatieganu University of Medicine and Pharmacy, Cluj-Napoca, Romania

The 12^th^ World Federation for Neurorehabilitation (WFNR) hybrid Congress, held between 14–17 December 2022 in Vienna (Austria), was a highly successful and intellectually stimulating event, bridging science and practical applications and offering insight into future avenues for neurorehabilitation (Figure 1). Attended by over 1000 on-site and 1500 online participants, the congress was chaired by Volker Hömberg (Neurologie – SRH Gesundheitszentrum, Germany) and Caterina Pistarini (Istituti Clinici Scientifici Maugeri IRCCS, Italy) featuring presentations from world-renowned experts in the field of neurorehabilitation.

**Figure 1:**
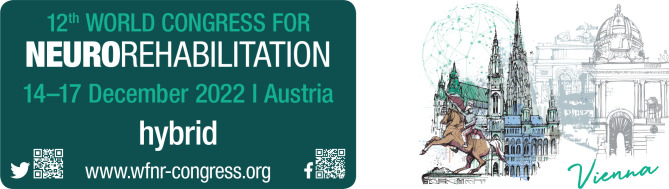
World Federation for Neurorehabilitation – 12^th^ World Congress for NeuroRehabilitation.

**The World Federation for Neurorehabilitation** (WFNR) is a multidisciplinary umbrella organization for national neurorehabilitation societies worldwide, with over 5.000 members, 38 Special Interest Groups (SIGs), and over 41 national societies affiliated around the globe. The WFNR is directed towards advancing research, education, and clinical practice in the domain of neurorehabilitation at a global level through providing training, encouraging collaboration and research, and offering a forum for professionals interested in neurorehabilitation. SIGs working across different disciplines promote the development of their specific area of interest within the general aims of WFNR with a focus on engagement in educational activities. During the WCNR 2022, a WFNR General Assembly was organized, and Professor Volker Hömberg became the active president of the federation. Moreover, the event also hosted 14 SIG meetings.

Set in the charming city of Vienna (Austria) in the wintertime, the event showcased a stimulating program focused on multidisciplinary approaches to neurosciences and neurorehabilitation from international acclaimed experts. The 2022 WFNR World Congress for Neurorehabilitation covered topics related to stroke, spasticity, epilepsy, neurophysiology, neuromuscular disorders, Parkinson's disease, multiple sclerosis, spinal cord injury, autonomic dysfunction, disorders of consciousness, public health, traumatic brain injury, neuromodulation, pain management, neuroimaging, outcomes research, brain-computer interfaces and more. The rich and varied program allowed attendees to explore in depth the rapidly growing field of neuroscience and neurorehabilitation and provided a forum for the exchange of ideas and knowledge.

The Organizing Committee consisted of well-established international names from the area of neurosciences and neurorehabilitation, including Volker Hömberg (Germany), Dafin Muresanu (Romania), David Good (USA), Caterina Pistarini (Italy), Leonard Li (Hong Kong), Jorge Hernández Franco (Mexico), Teresita Joy Evangelista (Philippines), Nirmal Surya (India), Sabahat Wasti (United Arab Emirates), Lucia Braga (Brazil), Stephanie Clarke (Switzerland), Arseny Sokolov (Switzerland), Adrian Guggisberg (Switzerland), and Heinrich Binder (Austria). The Scientific Program Committee was comprised of Caterina Pistarini (Italy), Volker Hömberg (Germany), Gilles Rode (France), Dominic Pérennou (France), Janice Eng (Canada), Catherine Lang (USA), Randolph Nudo (USA), Thomas Platz (Germany), Nam-Jong Paik (Republic of Korea), Wayne Feng (USA), Abhishek Srivastava (India), Adrian Guggisberg (Switzerland), Thomas Nyffeler (Switzerland), Arseny Sokolov (Switzerland), and Michael Thaut (Canada).

## Scientific Perspectives – advancing knowledge and education worldwide through multidisciplinary approaches

Surjo R. Soekadar (Germany) delivered the Michael P. Barnes Lecture, focusing on the recognition of the visionary leadership and dedication of the founding WFNR President. Michael Chopp (USA), Janice Eng (Canada), Pam Enderby (UK), and Katrin Amunts (Germany) subsequently delivered key lectures on neurovascular restorative and protective therapies for cerebrovascular disease, evidence-based neurological rehabilitation in stroke, speech and language therapy, the role of microRNA in neural repair, and brain mapping.

Special lectures were offered by two world-renowned in the domain of neuroscience:

- “The concept of neurobiological reserve and the impact on stroke recovery” – Prof. Dafin Fior Muresanu (Romania)
- “The comprehensive, customized, cost-effective approach (CCCAP) to prevention of dementia” – Prof. Vladimir Hachinski (Canada)

Inspiring joint sessions were organized in collaboration with several related societies, pinpointing the intercultural and interdisciplinary approach. The collaborative symposia have been held in collaboration with the Foundation of the Society for the Study of Neuroprotection and Neuroplasticity (SSNN), ICF Education, World Federation of Neurology (WFN), World Stroke Organization (WSO), Cochrane Rehabilitation, and the Academy for Multidisciplinary Neurotraumatology (AMN).

Furthermore, the congress brought together:

- 1 pre-Congress teaching course on stroke rehabilitation practice recommendations with 14 modules;
- e-Poster Sessions;
- 5 plenary lectures;
- Workshops;
- A dedicated session for Young Clinicians (Thematically coordinated sessions targeting junior investigators);
- 46 symposiums and 7 joint symposiums;
- 6 oral communication sessions;
- 2 round tables;
- 3 Developing World Forums;
- 2 Ask me! Session;
- 1 Career development session (Neurorehabilitation career symposium);
- 14 Poster Sessions.

**Figure 2:**
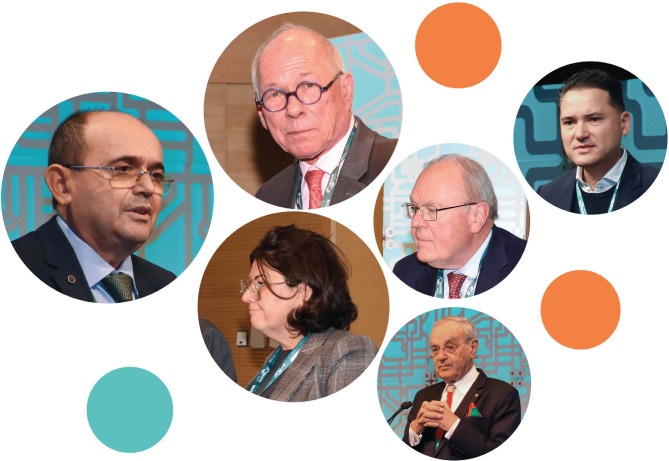
World-renowned experts from the WCNR 2022.

The program was designed to bring together science and practical applications, with the WCNR experts (Figure 2) offering insight into future avenues for neurorehabilitation. The interactive sessions provided attendees with the opportunity to engage with experts and learn from their vast experience. A key trend that emerged from the congress was the growing importance of interdisciplinary collaboration to address the complexity of neurological conditions based on a multidisciplinary approach to treatment. Moreover, the experts emphasized the need for close collaboration among different healthcare professionals, patients, and their families. Another area of focus was the need for better measurement of outcomes and the development of more sensitive and reliable tools for assessing the effectiveness of neurorehabilitation interventions. This is particularly important considering the wide range of neurological conditions and the varying responses to treatment.

A series of interviews with top-notch experts from the WCNR were carried out by the WFNR and the EFNR Teams, with recordings of the interviews to be further released on the EFNR website. An adapted interview series will soon be available to readers, starting January 2023, on the Journal of Medicine and Life website.

## Industry Insight – following the most exciting technological and medical developments in neurorehabilitation

Industry sessions and workshops were presented during the event. Exhibitions from industry partners were available for the participants to explore new technological advancements in neurorehabilitation, such as Virtual and Augmented Reality, rehabilitation robots for upper and lower limbs, stroke therapy, eye-tracking technologies, bi-manual mirror training, intensive and early mobilization, brain plasticity via intensive visual stimulation, occupational therapy, kinesiotherapy & virtual walk, functional electrical stimulation, and automated simulation of brain stimulation.

Overall, the 12^th^ World Federation for Neurorehabilitation Congress was a valuable and informative event, bringing together leading researchers and practitioners in neuroscience and neurorehabilitation. The event provided a forum for the exchange of ideas and knowledge and offered a glimpse into the latest research and developments in the field. We look forward to the continued growth and advancement of the domain of neurorehabilitation in the coming years and its positive impact on the lives of individuals with neurological conditions.

**Figure 3:**
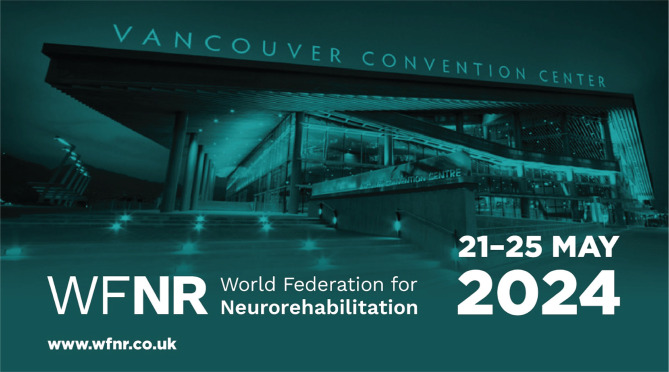
World Federation for Neurorehabilitation – 2024 Congress, Vancouver (Canada).

The next WFNR Congress will take place in 2024 in Vancouver, Canada, to further new avenues for neurorehabilitation research, education, and practical applications and encourage specialists worldwide to collaborate towards improving the fields of neuroscience and neurorehabilitation (Figure 3). The congress will be a hybrid event, allowing attendees worldwide to participate and engage with the content, regardless of location.

So, to get a glimpse into the intricacies of neurotrauma and neurorehabilitation and contribute to the advancement of neuroresearch and neurorehabilitation, join us for the 13^th^ WFNR Congress in 2024 in Vancouver, Canada!

**WFNR: Advancing research, education, and clinical practice in neurorehabilitation throughout the world!**

